# Supplementary material for: Is there genetic variation in mycorrhization of Medicago truncatula?
Source: PeerJ. 2017 Sep 7;5:e3713. doi: 10.7717/peerj.3713 (PMC5592082; doi:10.7717/peerj.3713)
Supplement: Table S1 [file peerj-05-3713-s001.pdf]

Table S1: List of the 33 accessions analyzed in this study

Hapmap ID	Line No*	Population of origin (accession)	Country of origin	Category
HM000 (HM101)	A17_Varma	A17	NA	Doug Cook UC Davis
HM001	L000163	SA22322	Syria	CC8
HM002	L000174	SA28064	Cyprus	CC8
HM003	L000544	ESP105-L	Spain	CC8
HM004	L000736	DZA045-6	Algeria	RILParent/CC8
HM005	L000734	DZA315-16	Algeria	RILParent/CC8
HM006	L000530	F83005-5	France	RILParent/CC8
HM007	L000651	Salses71B	France	CC8
HM008	L000368	DZA012-J	Algeria	CC8
HM009	L000555	GRC020-B	Greece	CC16
HM010	L000154	SA24714	Italy	CC16
HM011	L000543	DZA327-7	Algeria	CC16
HM012	L000239	SA26063	Morocco	CC16
HM013	L000648	Salses42B	France	CC16
HM014	L000542	DZA233-4	Algeria	CC16
HM015	L000550	F11013-3	France	CC16
HM016	L000049	SA09707	Tunisia	CC16
HM031	L000545	ESP158-A	Spain	CC32
HM032	L000549	F11005-E	France	CC32
HM033	L000552	F20047-A	France, Corsica	CC32
HM034	L000554	F20089-B	France, Corsica	CC32
HM035	L000679	F66017	France	CC32
HM036	L000337	GRC042-1	Greece	CC32
HM037	L000557	GRC064-B	Greece	CC32
HM038	L000369	PRT180-A	Portugal	CC32
HM039	L000263	SA03116	Israel	CC32
HM040	L000321	SA03780	Italy	CC32
HM041	L000198	SA09048	Libya	CC32
HM042	L000290	SA09119	Turkey	CC32
HM043	L000310	SA09944	Tunisia	CC32
HM044	L000245	SA14161	Jordan	CC32
HM045	L000144	SA14163	Jordan	CC32
HM046	L000213	SA27882	Morocco	CC32

*All line numbers used in the text were abbreviated and contained the last three numerals only.
