# Supplementary material for: Is there genetic variation in mycorrhization of Medicago truncatula?
Source: PeerJ. 2017 Sep 7;5:e3713. doi: 10.7717/peerj.3713 (PMC5592082; doi:10.7717/peerj.3713)
Supplement: Table S2 [file peerj-05-3713-s002.pdf]

Table S2: Summary of two-factorial ANOVA comparing ecotypes and treatments (shown in Fig. 1)

| | Df | Sum Sq | Mean Sq | F value | Pr (>F) |
|---|---|---|---|---|---|
| Factor 1 (ecotypes) | 32 | 16128 | 504 | 1.856 | 0.00477 ** |
| Factor 2 (treatments) | 3 | 133637 | 44546 | 164.033 | < $2e^{-16}$ *** |
| Factor1:Factor2 | 96 | 22796 | 237 | 0.874 | 0.77639 |
| Residuals | 263 | 71421 | 272 | | |
