# Supplementary figures and images for: Is there genetic variation in mycorrhization of Medicago truncatula?
Source: PeerJ. 2017 Sep 7;5:e3713. doi: 10.7717/peerj.3713 (PMC5592082; doi:10.7717/peerj.3713)

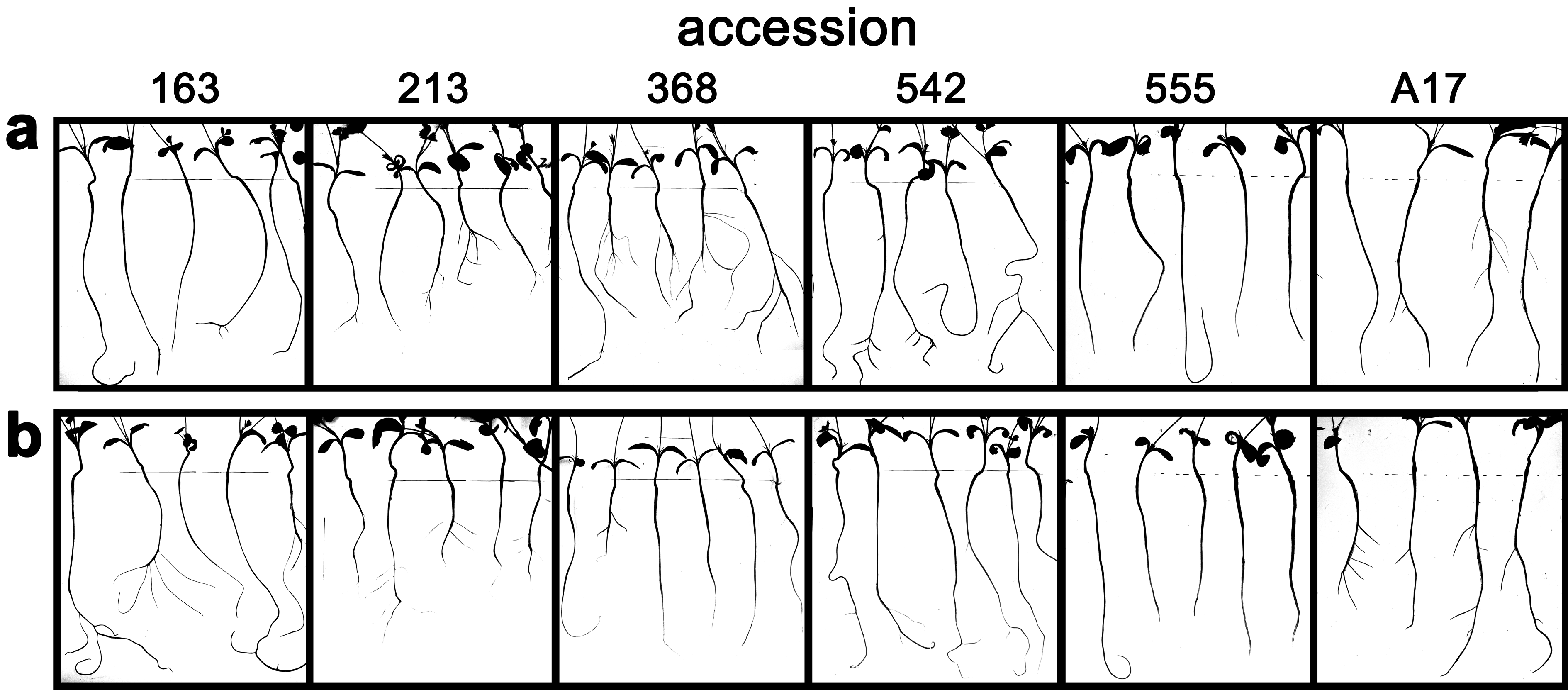

Supplement: Figure S1 — Seedlings were grown on plates containing either 100 µM Pi (A) or 3 µM Pi (B). Pictures were taken seven days after transfer of seedlings to plates. [file peerj-05-3713-s003.png]

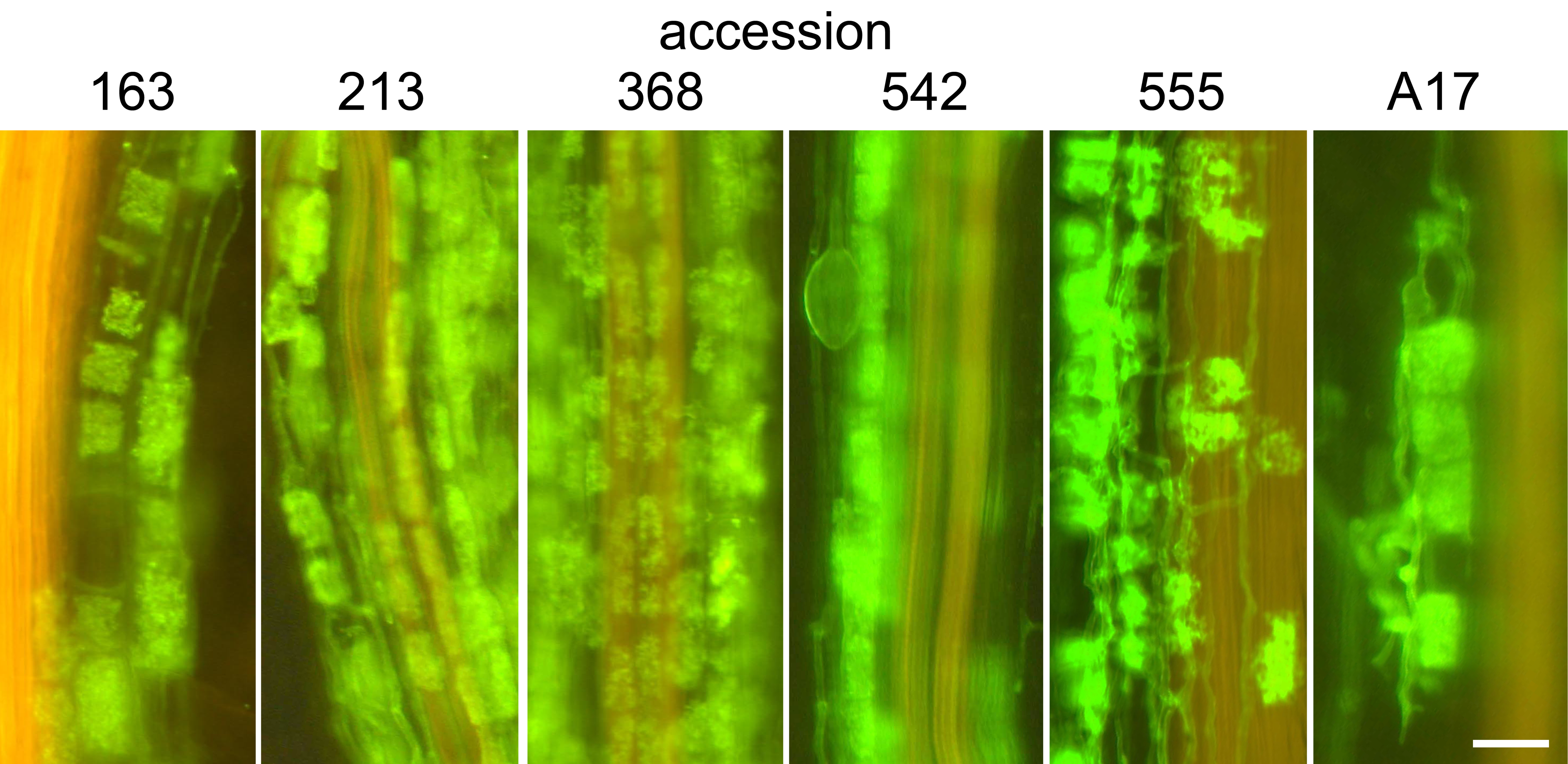

Supplement: Figure S2 — Roots of plants inoculated with R. irregularis for two weeks under full Pi supply were stained with WGA-AlexaFluor488. Bar represents 50 µm for all micrographs. [file peerj-05-3713-s004.png]

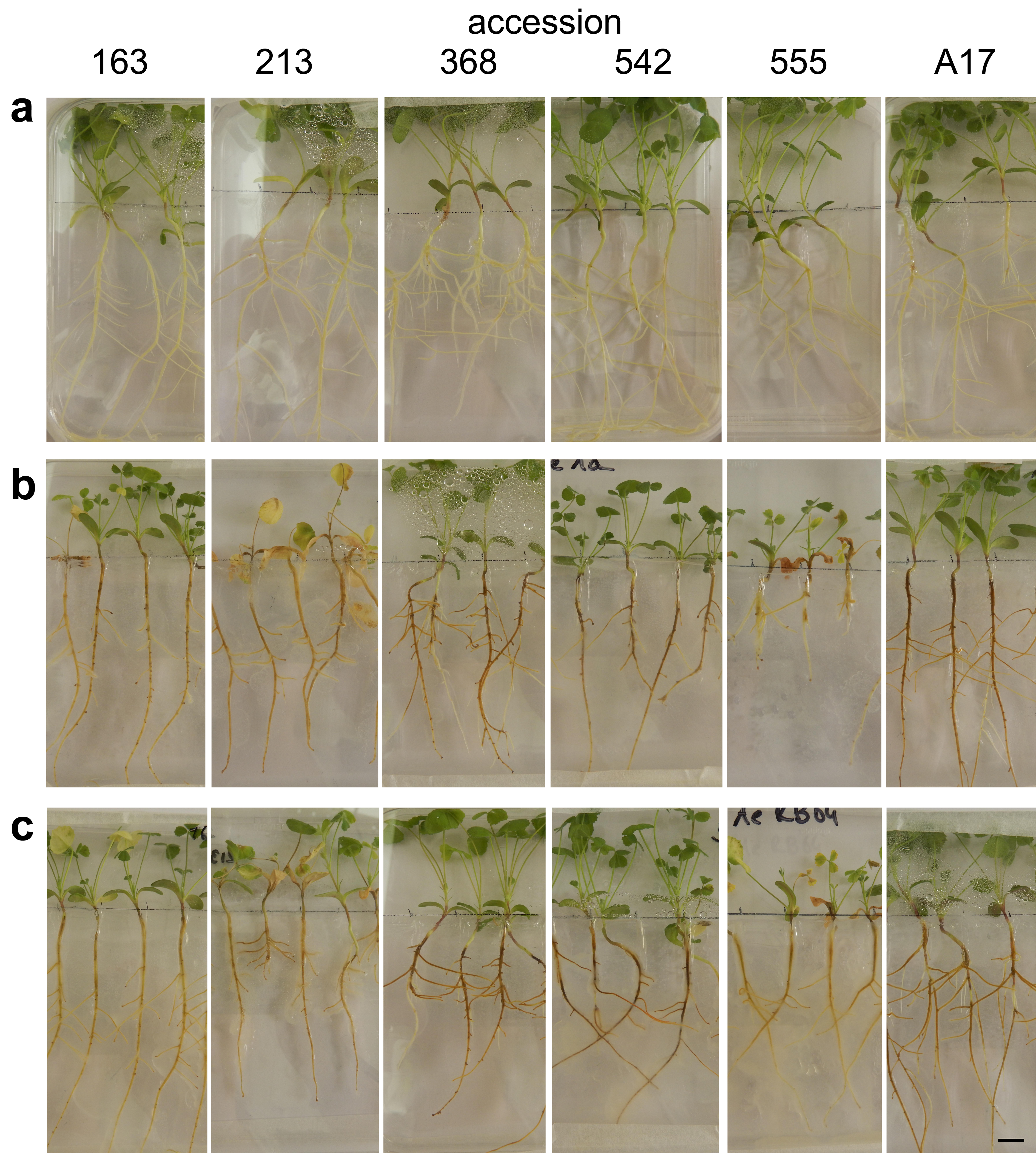

Supplement: Figure S3 — Seedlings of selected accessions were cultivated on vertical plates and infected either with A. euteiches strain GB I1 (B) or strain AERB84 (C). Non-infected controls are given in (A). Bar represents 1 cm for all photographs. [file peerj-05-3713-s005.png]

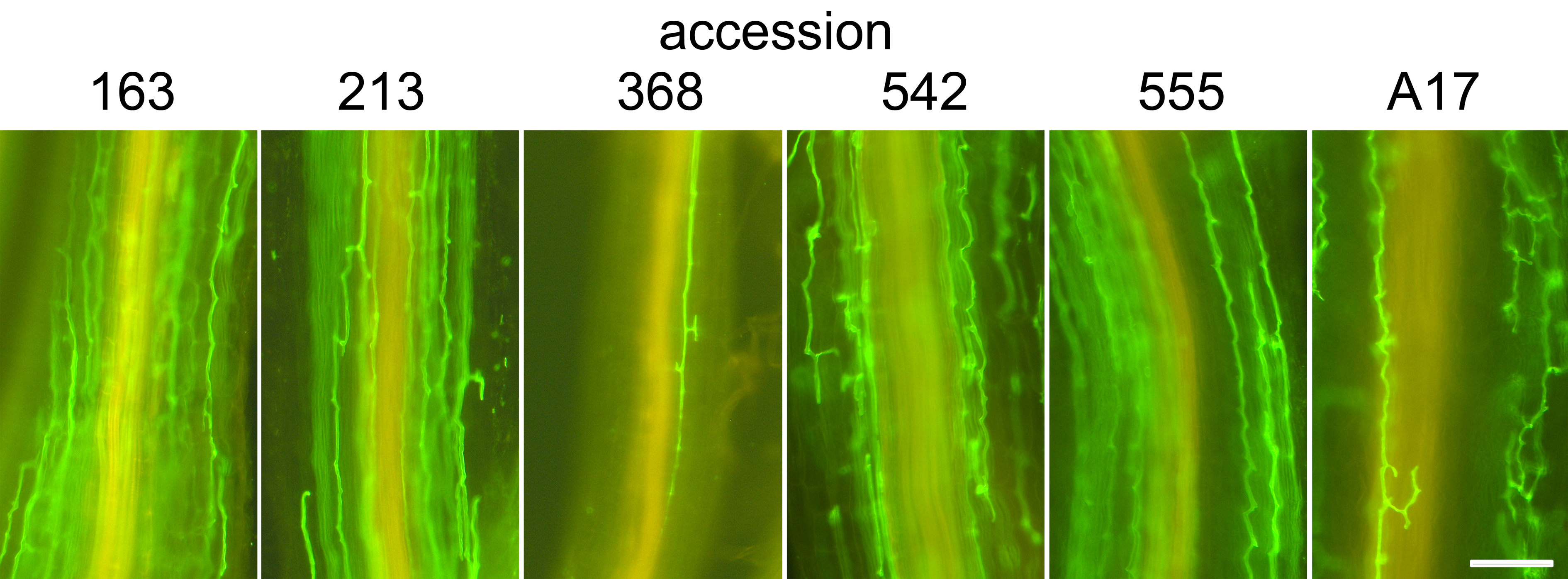

Supplement: Figure S4 — Roots of plants grown in expanded clay and infected with A. euteiches strain GB I1 for four weeks were stained with WGA-AlexaFluor488. Bar represents 100 µm for all micrographs. [file peerj-05-3713-s006.png]
